# Physical therapy for sleep apnea: a smartphone application for home-based physical therapy for patients with obstructive sleep apnea

**DOI:** 10.3389/fneur.2023.1124059

**Published:** 2023-05-25

**Authors:** Khue Bui-Diem, Ching-Hsia Hung, Guan-Cheng Zhu, Nguyen Van Tho, Thu Nguyen-Binh, Quan Vu-Tran-Thien, Duy To-Truong, Hoan Ngo-Thanh, Sy Duong-Quy

**Affiliations:** ^1^Department of Physiology - Pathophysiology - Immunology, University of Medicine and Pharmacy at Ho Chi Minh City, Ho Chi Minh City, Vietnam; ^2^Department of Physical Therapy, National Cheng Kung University, Tainan, Taiwan; ^3^Department of Tuberculosis and Lung Diseases, University of Medicine and Pharmacy at Ho Chi Minh City, Ho Chi Minh City, Vietnam; ^4^Department of Orthopaedics and Rehabilitation, University of Medicine and Pharmacy at Ho Chi Minh City, Ho Chi Minh City, Vietnam; ^5^School of Biomedical Engineering, International University, Vietnam National University - Ho Chi Minh City, Ho Chi Minh City, Vietnam; ^6^Sleep Lab Center, Lam Dong Medical College, Dalat, Vietnam; ^7^Hershey Medical Center, Penn State Medical College, State College, PA, United States

**Keywords:** obstructive sleep apnea, home-based physical therapy, smartphone application, physical therapy, respiratory muscle training

## Abstract

**Purpose:**

In this study, we described “PT for Sleep Apnea”, a smartphone application for home-based physical therapy of patients with Obstructive Sleep Apnea (OSA).

**Methods:**

The application was created in a joint program between the University of Medicine and Pharmacy at Ho Chi Minh City (UMP), Vietnam, and National Cheng Kung University (NCKU), Taiwan. Exercises maneuvers were derived from the exercise program previously published by the partner group at National Cheng Kung University. They included exercises for upper airway and respiratory muscle training and general endurance training.

**Results:**

The application provides video and in-text tutorials for users to follow at home and a schedule function to assist the user in organizing the training program, which may improve the efficacy of home-based physical therapy in patients with Obstructive Sleep Apnea.

**Conclusion:**

In the future, our group plans to conduct a user study and randomized-controlled trials to investigate whether our application can benefit patients with OSA.

## 1. Introduction

Obstructive sleep apnea (OSA) is a type of sleep-related breathing disorder in the adult population. The operational definition of OSA is repeated upper-airway collapse and narrowing-induced apnea/hypopnea during sleep (1). Current estimates indicate that OSA affects 10–30% of the adult population worldwide, with higher prevalence in the male and the aging/aged population (1). The direct cause of apnea and hypopnea in OSA is the repeated collapse or narrowing of the upper airway during sleep. Thus, in theory, the problem of apnea/hypopnea during sleep can be mitigated by preventing upper airway collapse. Recent studies suggest multiple factors contribute to the collapse of the upper airway during sleep, including anatomical and non-anatomical factors (2). Anatomical factors include excessive fat accumulation at the tongue base and neck muscles, having a longer upper airway, or having larger tonsils and adenoids (3–7). Whereas non-anatomical factors included weakness and inadequate responsiveness of intrinsic/extrinsic muscles of the tongue, low arousal threshold, or unstable respiratory control (8).

Current clinical management of OSA includes continuous positive airway pressure (CPAP), trans-oral surgery to remove excess soft tissue, mandibular advancement device (MAD), and oral-pharyngeal physical therapy (2). In these, CPAP is the most effective in preventing upper airway collapse regardless of the cause (2). However, the patient adherence to CPAP is less than optimal due to its cost and interference with sleep (2).

On the other hand, oral-pharyngeal physical therapy may mitigate the severity of sleep apnea by improving the tension, stiffness, and responsiveness of intrinsic/extrinsic muscles of the tongue and the muscles that control the movement of the soft palate, thus preventing the collapse of the upper airway (9–14). Our previous study has shown that a 12-week, hospital-based physical therapy program can significantly alleviate the symptoms of OSA (15). However, a significant limitation of hospital-based exercise is that the patient needs to physically move from home to the hospital, which may involve many factors that ultimately affect the patients' adherence (16). For example, patients with low mobility and social support may have no one to transport them to the hospital once a week to partake in the rehabilitation program. For various reasons, a home-based rehabilitation program is essential to many rehabilitation programs (17). Recent studies also showed that home-based rehabilitation programs could be as effective as one-on-one rehabilitation with a physical therapist (18–22). In addition, during the COVID-19 pandemic visiting the hospital to attend a rehabilitation program may be unnecessary in many places (23). However, a significant challenge of the home-based rehabilitation program is that the patient needs to perform the exercise without the on-site instruction of a physical therapist. Thus, in this study, we designed a smartphone application to provide step-by-step instruction and guidance to assist patients with OSA in performing home-based physical therapy programs.

## 2. Materials and methods

### 2.1. Exercise maneuvers used in this study

This smartphone application is created in a joint research program between the University of Medicine and Pharmacy at Ho Chi Minh City (UMP), Vietnam, and National Cheng Kung University (NCKU), Taiwan. This application is the first step in the study “Smartphone application of physical therapy for obstructive sleep apnea patients”. Exercises maneuvers demonstrated in this application were derived from the exercise program previously published by the partner group at National Cheng Kung University (15). The exercise program included two parts: a portion of the upper airway and respiratory muscle exercise and a portion of general endurance exercise. The upper airway and respiratory muscle exercise program aimed to improve muscle strength and muscle tone for the intrinsic and extrinsic tongue muscles and respiratory muscles. Whereas general endurance exercises were directed at improving muscle tone and mobility of pharyngeal and soft tissue to improve airway closure during sleep. This program also aims at reducing accumulative fat in the oropharynx. A detailed description of the exercise program used in this study is summarized in the Supplementary material.

### 2.2. Architectural design

This app was designed by Medical AI Co., Ltd., Vietnam, with ideas and corrections by the UMP team. Currently, the language in this app is Vietnamese, and it is a prototype. It has been developed for the Android mobile operating system via Android Studio. This tool is an open-source platform initially introduced by Google as the official IDE for Android app development (24). Android Studio allows developers to design various creative applications written in the Java programming language. Android Studio includes perfect functions as well as libraries to develop an application, design user-interface, run emulators [“Android Virtual Devices (AVDs)], and build APK (Android Package) files easily. Furthermore, SQLite was chosen as a database server in this app, the most common database engine that is a serverless and self-contained database (25).

## 3. Results

### 3.1. User interface and contents

**Figure 1:**
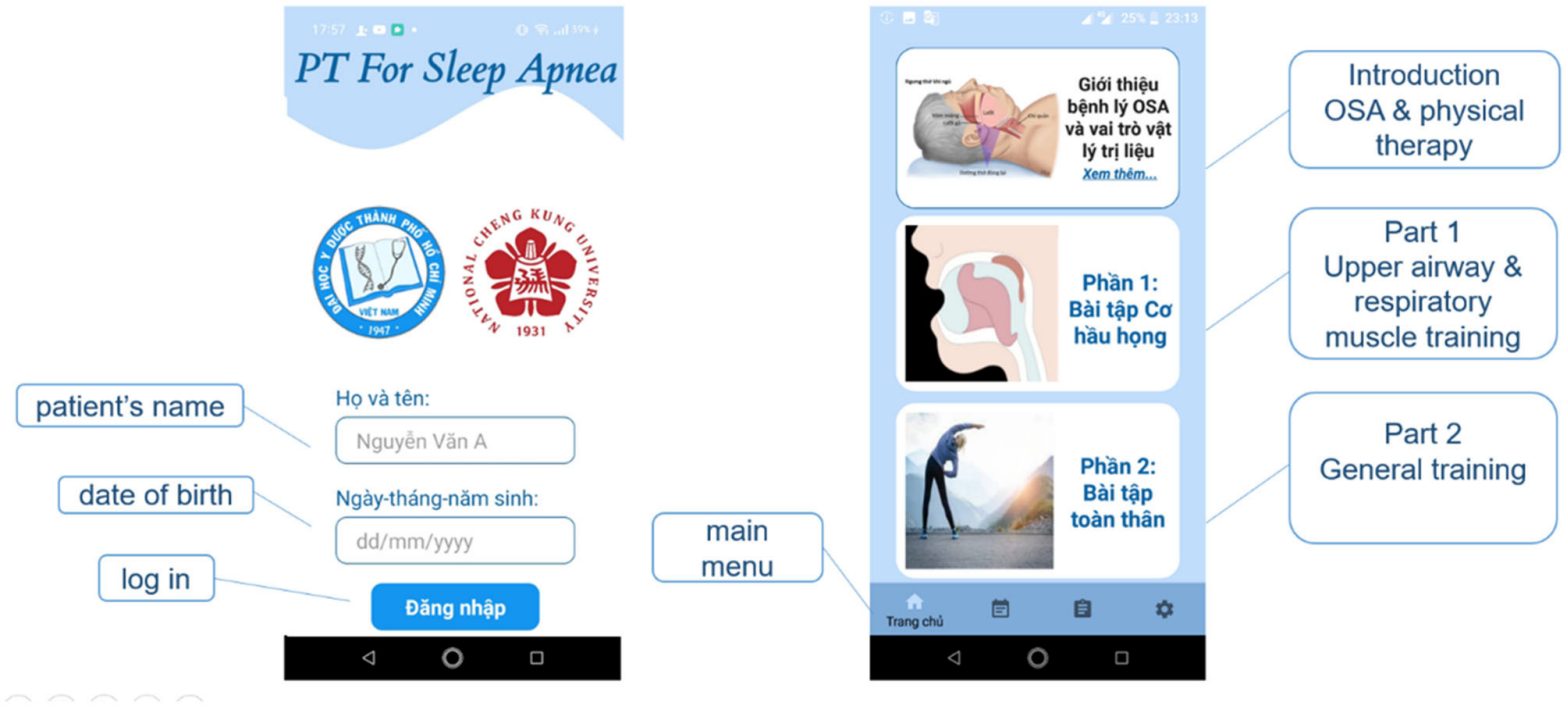
Log-in and home screen.

When the application starts, a login screen will ask the patients to fill in their Full name and “Date of Birth” login information. When the user enters the correct and sufficient information, then press the “Login” button to access the application's content. The toolbar at the bottom of the screen includes 4 main items: Home screen, Schedule, Diary, and Setting (Figure 1). The home screen (Figure 1) contains 3 main pieces of information: Introduction to OSA and the role of physiotherapy, tutorials for upper airway and respiratory muscle exercise, and tutorials for general endurance training.

The introduction to OSA and the role of physiotherapy shows the information about OSA and the role of physiotherapy in mitigating the severity of the disease.

**Figure 2:**
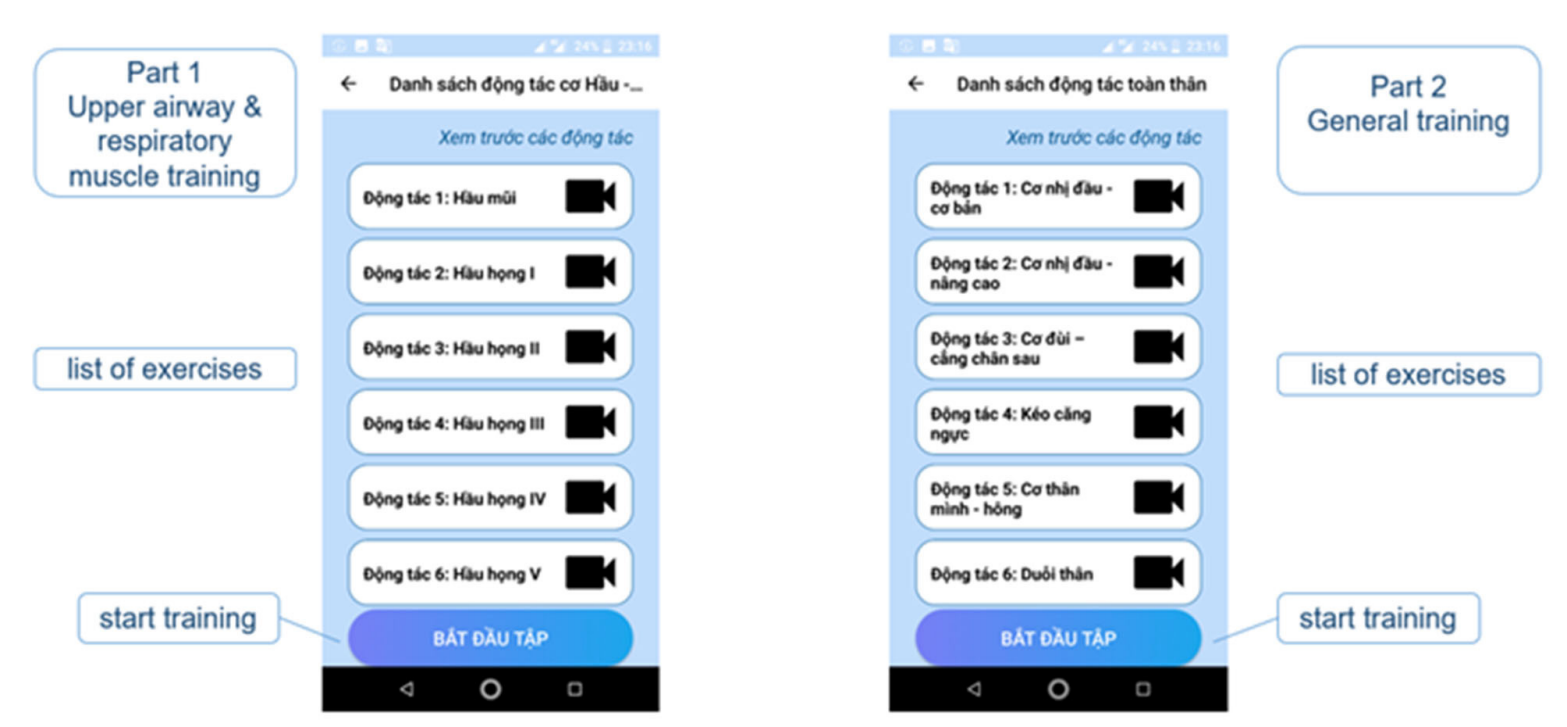
List of exercises.

**Figure 3:**
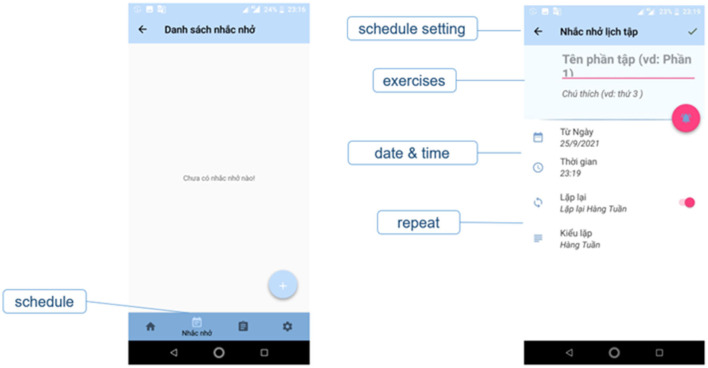
Schedule screen.

**Figure 4:**
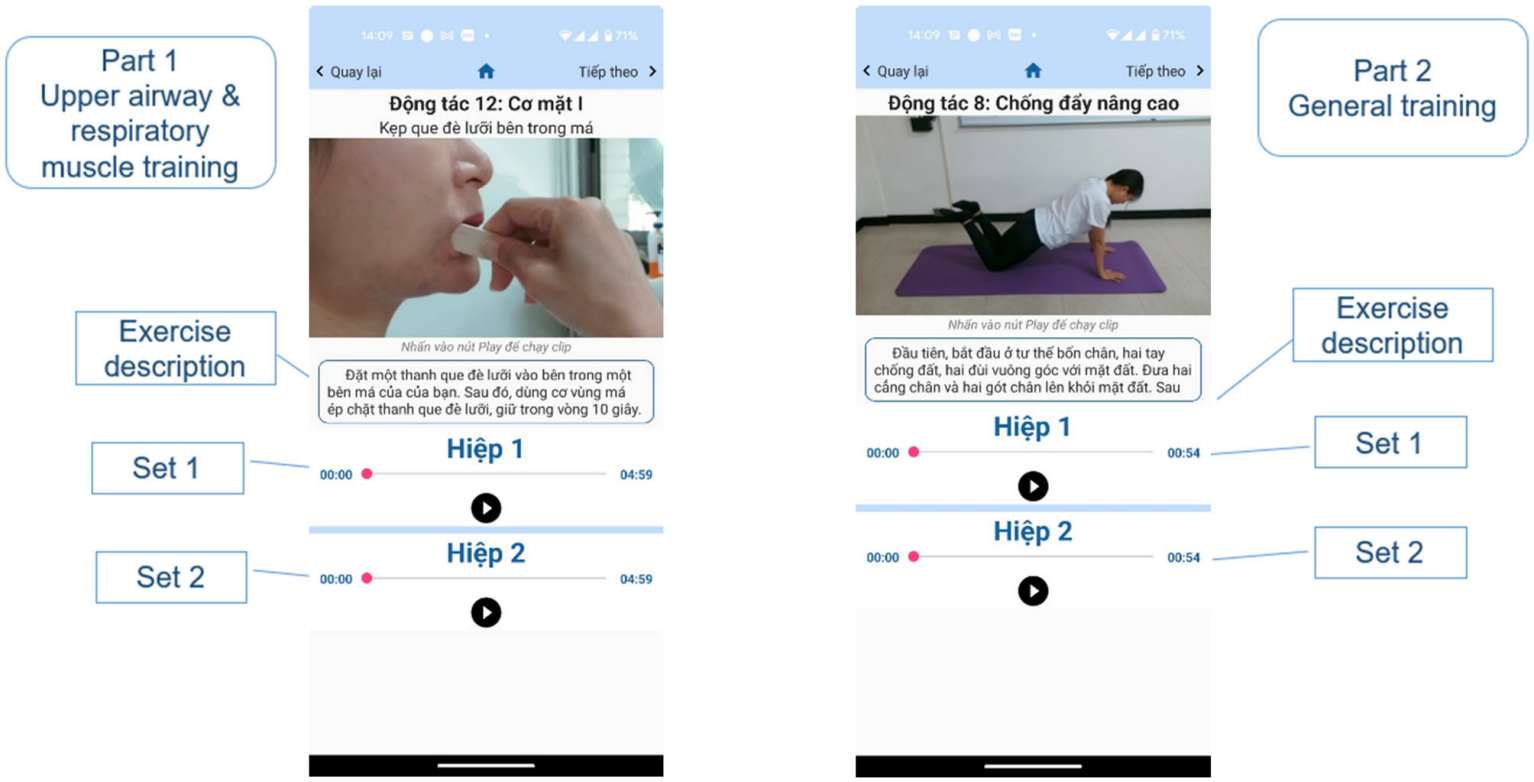
Training screen.

**Figure 5:**
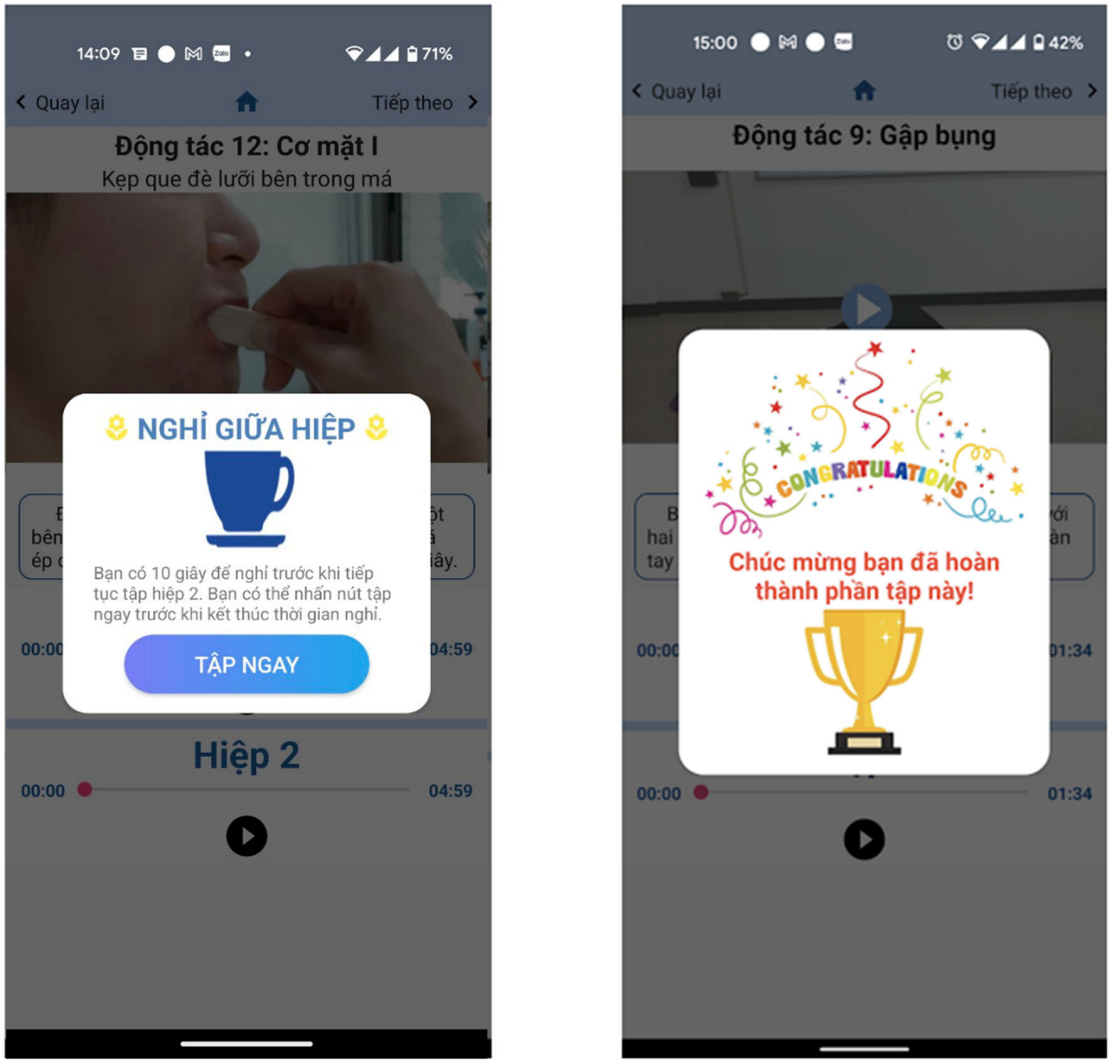
Screenshot of a break after set 1 and congratulations after finishing each part.

The upper airway and respiratory muscle exercise part includes the list of exercises in this part (Figure 2). The page contains 3 main sections; the first is the review, and users can view the videos to familiarize themselves with the instructions before starting the official training. Here, users can actively select the exercises they want to see in the list of exercises. The second is the list of the exercises, users can click on the exercises they want to practice, but encourage users not to practice randomly, but should practice in numbered order to ensure they complete the whole program. The third section is the actual exercise program. There is a “Start training” button: when the button is clicked, the program will start from the 1st exercise on the list, followed by the rest of the exercise program. There is also a schedule function to help users remember the exercise time to achieve optimal training efficiency (Figure 3). In each exercise, there will be a video tutorial and text description to remind the user how to perform that exercise, users can play or stop the video tutorial at any time (Figure 4). When the user pressed the start (▸) button, the application will play the video tutorials of each exercise by order of appearance. After finishing an exercise, there will be a 10–30 s break before the next exercise starts. When the user completes the whole set, a congratulations notice will pop up to congratulate the user (Figure 5).

The user interface for the general endurance exercise section is identical to the upper airway and respiratory muscle exercise section.

## 4. Discussion

The home-based rehabilitation program is becoming an essential part of many rehabilitation programs (17). It is even more so during the COVID-19 pandemic since for many places visiting the hospital for rehabilitation purposes may be considered an unnecessary risk (23). However, the nature of home-based rehabilitation dictates that the patient performs rehabilitation exercises without the supervision of a professional physical therapist or other medical professionals. Thus, the performance quality often relies solely on the patient's memory or the assisting family/caregiver, which is not an optimal approach for any treatment program. The use of smartphone applications for home-based rehabilitation programs has been a popular topic in the clinical community. Several studies have investigated the effect of smartphone applications on Parkinsonism, fall prevention, and exercise for the elderly (26–29). However, to the best of our knowledge, there had not been a program, device, or application designed to assist patients with OSA in performing rehabilitation programs at home. Compared to rehabilitation programs for Parkinson's disease or fall prevention, which consist mostly of gross motor skills, the rehabilitation program for OSA requires a level of dexterity (15, 27, 28). Thus, it is even more critical that the patients with OSA can correctly perform the rehabilitation program at home to improve their condition. Our smartphone application enables the patients to review the step-by-step instruction of the rehabilitation program anytime, anywhere, thus improving the effectiveness of the habilitation program.

This application is designed for obstructive sleep apnea patients, at all levels of this syndrome, especially for those who cannot afford or tolerate CPAP therapy, or it can be an adjunct therapy to CPAP. According to the American Academy of Sleep Medicine Scoring Manual of Polysomnography, older children (from 13 years old) can be used for OSA diagnosis criteria as adults, so they can also practice these exercises (30). As for the technical aspect, some elderly may have difficulty using a smartphone, but they can also use the application with the help of their caregivers. In short, our design and exercises can be suitable for patients from 13 years old.

There are some applications for sleep apnea to practice upper airway muscle strength, such as Airway Gym and SnoreGym. However, these applications do not include general endurance exercises and there are no real-person videos like our design. In our application, the instructions are described in both video and text, once the patients are used to it, they can simply look at the exercise name and text instructions. Another advantage of our design is the narration in each set automatically runs, eliminating the need for patients to look at their phones constantly.

Although the application is a training aid, there are also some notices: self-discipline to complete exercises according to instructions, practice regularly according to the protocol, record how to practice correctly, and contact a doctor when needed support. Currently, our app can partially support patient self-discipline and frequency through scheduling. The main limitation of the application is that it does not track the patient's movements, so the effect may be less than the direct exercises with the instructor. To achieve this tracking goal, we have the idea of using a phone camera when the patient is exercising, along with trained artificial intelligence to check the movement. However, this requires a large budget and time, so the research team has yet to be able to do it. In addition, the application design does not have a sleep tracking section (snoring and sleep-wake time), we will gradually develop it for the following versions. These limitations are challenges for telemedicine in general and smartphone apps in particular. In addition to the limitations mentioned above, our application also has the disadvantage that the design is not competitive, if it is designed as a game, the patients will be more motivated to exercise. These features will be studied in the future.

## 5. Conclusion

Our group plans to conduct a small-scale deployment and user study to investigate whether the application can effectively assist patients with OSA in performing their rehabilitation program at home. In addition, we also plan to conduct user interviews to understand what other functions the users would like to have in the application to further assist them in performing the rehabilitation program. Finally, we aim to achieve a large-scale randomized-controlled trial to investigate whether our application can benefit patients with OSA.

## Data availability statement

The original contributions presented in the study are included in the article/Supplementary material, further inquiries can be directed to the corresponding author.

## Ethics statement

This study was approved by the Institutional Review Board of the University of Medicine and Pharmacy at Ho Chi Minh City (UMP), Vietnam. Approval number: IRB-VN01002/IORG0008603/FWA00023448. The patients/participants provided their written informed consent to participate in this study.

## Author contributions

KB-D, C-HH, G-CZ, and SD-Q conceived the research idea and contributed to the final manuscript. KB-D, TN-B, and DT-T practiced the original PT exercises to design app flow, adjusted the commands and break time to suit the app version, and discussed ideas with the coding and design team. NT and QV-T-T advised on the protocol, the app, and OSA syndrome. DT-T discussed with the NCKU team about the original PT exercises and translated the exercises into Vietnamese. HN-T discussed ideas with the coding and design team and edited the final manuscript. All authors contributed to the article and approved the submitted version.
