# Supplementary material for: Physical therapy for sleep apnea: a smartphone application for home-based physical therapy for patients with obstructive sleep apnea
Source: Front Neurol. 2023 May 25;14:1124059. doi: 10.3389/fneur.2023.1124059 (PMC10249728; doi:10.3389/fneur.2023.1124059)
Supplement: Supplementary file 1 [file Data_Sheet_1.PDF]

Supplementary Material

Physical Therapy for Sleep Apnea: A smartphone Application for Home-Based Therapy for Patients with Obstructive Sleep Apnea

Khue Bui-Diem[1], Ching-Hsia Hung[2], Guan-Cheng Zhu[2], Tho Nguyen-Van[3], Thu Nguyen-Binh[1], Quan Vu-Tran-Thien[1], Duy To-Truong[4], Hoan Ngo-Thanh[5], Sy Duong-Quy[6,7*]

*** Correspondence:** Sy Duong-Quy, sduongquy.jfvp@gmail.com

Table: Description of Exercises

No.	Part	Exercise's name	Description	Time on the app (including command time)	
				**minute**	**second**
1	PART 1: Upper airway & Respiratory muscle exercise	Retropalatal Exercise	• Open your mouth<br>• Pronounce "A" for 5 seconds<br>• Stop. Close your mouth<br>• Repeat that movement 10 times per set<br>• And perform 2 sets per session	1	32
2		Retroglossal exercise I	• Push the tongue against the front of the palate<br>• And move it back to perform a "click" sound<br>• Repeat that movement 10 times per set, and perform 2 sets per session	0	38
3		Retroglossal exercise II	• The patient will move their tongue<br>• From right to left and from up to down<br>• The sequence of movement directions can be randomized to increase the challenge for repetitions<br>• Perform 10 repetitions per set	1	8

			and perform 2 sets for each session		
4		Retroglossal exercise III	• In this exercise, the tongue of the patient will scroll<br>• Circle with their tongue on the inside of the lips<br>• Draw 10 circles per set.<br>• And perform 2 sets for each session.	1	8
5		Retroglossal exercise IV	• Forcing the back of the tongue against the floor of the mouth for 8 seconds<br>• Repeat the movement 10 times per set<br>• Perform 2 sets per session	2	2
6		Retroglossal exercise V	• Step 1, bite the tongue depressor.<br>• Step 2, push the tongue against the tongue depressor and hold for 8 seconds.<br>• Repeat that 10 times per set<br>• Perform 2 sets per session.	2	2
7		Deglutition Exercise I	• Gently holding the tongue between the front teeth<br>• Maitain this position<br>• Then, close your mouth and swallow<br>• Repeat that 10 times per set<br>• Perform 2 sets per session	1	8
8		Deglutition Exercise II	• This version is the same procedure as the beginner's version<br>• But swallowing while maintaining the neck in the extended position	1	8

			• Repeat that 10 times per set<br>• Perform 2 sets per session		
9		TMJ Exercise I.1	• Open your mouth against resistance<br>• Hold in 8 seconds<br>• Relax<br>• Repeat that 10 times per set<br>• Perform 2 sets per session	2	2
10		TMJ Exercise I.2	• Hold the tongue depressor by the front teeth<br>• Close your mouth against the resistance of the tongue depressor<br>• Maintain this position for 8 seconds.<br>• Relax<br>• Repeat that 10 times per set<br>• Perform 2 sets per session	2	2
11		TMJ Exercise II	• Protrude the jaw and hold for 8 seconds<br>• Relax<br>• Then, retrude the jaw and hold for 8 seconds<br>• Relax<br>• Repeat that 10 times per set<br>• Perform 2 sets per session	4	11
12		Facial Exercise I	• Put a tongue depressor inside the mouth<br>• Then, press the cheek against the tongue and hold for 10 seconds<br>• Relax<br>• Repeat that 10 times per set<br>• Perform 2 sets per session	4	59
13		Facial Exercise II	• Pucker the lips as if about to kiss<br>• Hold for 10 seconds<br>• Relax<br>• Repeat that 10 times per set<br>• Perform 2 sets per session	2	22

14		Facial Exercise III	• Make a balloon with your mouth while putting pressure on the cheek with your finger<br>• Hold it for 15 seconds<br>• Relax<br>• Repeat that 10 times<br>• And right cheek for 10 times in each set<br>• Perform 2 sets per session	6	39
15		Respiratory muscle (inhalation)	The equipment needed for these exercises includes 1, a straw. And 2. A glass filled with water<br>• Hold the straw with your hand and mouth as if smoking a pipe.<br>• Plug the open end of the straw with your finger.<br>• Inhale with your mouth against the resistance, and hold for 5 seconds.<br>• Repeat this maneuver 10 times per set, and perform 2 sets for each session.	1	32
16		Respiratory muscle (exhalation)	The equipment needed for these exercises includes 1. A straw, and 2. A glass filled with water.<br>• First, fill the glass with 10 centimeters of water. Then put the straw to the bottom of the glass.<br>• Exhale to the straw and continue exhaling for 5 seconds.<br>• Repeat this maneuver 10 times per set, and perform 2 sets for each session.	1	32
17	PART 2: General endurance exercise	Biceps stretch I.1 (basic)	• To stretch the biceps muscles<br>• The patient will extend her shoulder and elbow joints<br>• Maintain this position for 10 seconds<br>• Relax<br>• Repeat that 8 times per set<br>• Perform 2 sets per session	1	56

18		Biceps stretch I.2 (advanced)	• To further stretch the biceps muscle, the patient can bend forward while stretching<br>• Maintain this position for 10 seconds<br>• Relax<br>• Repeat that 8 times per set<br>• Perform 2 sets per session	2	14
19		Calf Stretch	• The patient will start in a standing position and take a step forward<br>• Then, try to touch the toe on that foot<br>• Maintain this position for 10 seconds<br>• And return to the starting position<br>• For the other side, do the same<br>• Repeat that 8 times per set<br>• Perform 2 sets per session	5	10
20		Chest Stretch	• The movement of chest stretch is similar to weightlifting<br>• At a starting position, put the fist around the height of the shoulder<br>• And then extended out already here<br>• Maintain this position for 10 seconds<br>• Relax<br>• Repeat that 8 times per set<br>• Perform 2 sets per session	1	56
21		Flexors Stretch	• For this exercise, the patient starts in a standing position<br>• Take a step forward and raise the hand above your head<br>• Maintain this position for 10 seconds and return to the starting position<br>• For the other side, the procedure is the same<br>• Repeat that 8 times per set<br>• Perform 2 sets per session	5	10

22		Trunk Stretch	• The patient starts in a kneeling sitting position<br>• And bent forward towards the crown<br>• Reach forward with your hand as far as possible while extending the back and neck<br>• Maintain that position for 10 seconds<br>• And then return to the starting position<br>• Relax<br>• Repeat that 8 times per set<br>• Perform 2 sets per session	2	54
23		M.pushups (basic)	• If the patient cannot perform push-up in the previous position<br>• The patient can push up in all fours position<br>• Repeat that 8 times per set<br>• Perform 2 sets per session	0	54
24		M.pushups (advanced)	• For this exercise, the patient starts in all fours position<br>• With the leg lifted over the ground<br>• Do push-ups<br>• Repeat that 8 times per set<br>• Perform 2 sets per session	0	54
25		Situp	• Start with a supine position with the knee flexed<br>• Make a cross across your chest with your hands<br>• Then, sit up and try to touch your knee with your elbows<br>• Repeat that 8 times per set<br>• Perform 2 sets per session	1	34
